# Loss of DJ-1 Does Not Affect Mitochondrial Respiration but Increases ROS Production and Mitochondrial Permeability Transition Pore Opening

**DOI:** 10.1371/journal.pone.0040501

**Published:** 2012-07-09

**Authors:** Emilie Giaime, Hiroo Yamaguchi, Clement A. Gautier, Tohru Kitada, Jie Shen

**Affiliations:** Center for Neurologic Diseases, Brigham and Women’s Hospital, Program in Neuroscience, Harvard Medical School, Boston, Massachusetts, United States of America; Genentech, United States of America

## Abstract

**Background:**

Loss of function mutations in the *DJ-1* gene have been linked to recessively inherited forms of Parkinsonism. Mitochondrial dysfunction and increased oxidative stress are thought to be key events in the pathogenesis of Parkinson’s disease. Although it has been reported that DJ-1 serves as scavenger for reactive oxidative species (ROS) by oxidation on its cysteine residues, how loss of DJ-1 affects mitochondrial function is less clear.

**Methodology/Principal Findings:**

Using primary mouse embryonic fibroblasts (MEFs) or brains from *DJ-1*−/− mice, we found that loss of DJ-1 does not affect mitochondrial respiration. Specifically, endogenous respiratory activity as well as basal and maximal respiration are normal in intact *DJ-1*−/− MEFs, and substrate-specific state 3 and state 4 mitochondrial respiration are also unaffected in permeabilized *DJ-1*−/− MEFs and in isolated mitochondria from the cerebral cortex of *DJ-1*−/− mice at 3 months or 2 years of age. Expression levels and activities of all individual complexes composing the electron transport system are unchanged, but ATP production is reduced in *DJ-1*−/− MEFs. Mitochondrial transmembrane potential is decreased in the absence of DJ-1. Furthermore, mitochondrial permeability transition pore opening is increased, whereas mitochondrial calcium levels are unchanged in *DJ-1*−/− cells. Consistent with earlier reports, production of reactive oxygen species (ROS) is increased, though levels of antioxidative enzymes are unaltered. Interestingly, the decreased mitochondrial transmembrane potential and the increased mitochondrial permeability transition pore opening in *DJ-1*−/− MEFs can be restored by antioxidant treatment, whereas oxidative stress inducers have the opposite effects on mitochondrial transmembrane potential and mitochondrial permeability transition pore opening.

**Conclusions/Significance:**

Our study shows that loss of DJ-1 does not affect mitochondrial respiration or mitochondrial calcium levels but increases ROS production, leading to elevated mitochondrial permeability transition pore opening and reduced mitochondrial transmembrane potential.

## Introduction

Parkinson’s disease (PD) is a neurodegenerative disorder characterized neuropathologically by the selective loss of dopaminergic (DA) neurons and the presence of Lewy bodies in the substantia nigra. Although most PD cases are sporadic, mutations in *parkin* (PARK2), *DJ-1* (PARK7) and *PINK1* (PARK6) have been linked to recessively inherited forms of parkinsonism [1], [2], [3]. Mitochondrial dysfunction, increased oxidative stress and dopaminergic dysfunction have been proposed as potential mechanisms underlying or contributing to dopaminergic neuronal degeneration [4], [5].

DJ-1 was originally cloned independently as a novel oncogene [6], a protein involved in fertilization [7] and a regulatory subunit of an RNA-binding protein complex [8], before it was associated with autosomal recessive forms of parkinsonism [2]. Subsequent studies discovered that DJ-1 is oxidized upon exposure to reactive oxidative species (ROS) [9], [10], and that oxidation occurs at three cysteine residues [11], [12]. While DJ-1 knockdown by siRNA or DJ-1 deficiency heightened their sensitivity to oxidative stress in a variety of model systems, including cell lines, embryonic stem cells, fruit flies and rodents [13], [14], [15], [16], [17], [18], overexpression of wild-type DJ-1 but not PD associated mutants protects cells against oxidative insult or mitochondrial toxins in these models [11], [14], [19], [20]. Under physiological conditions DJ-1 is localized mostly in the cytoplasm and the nucleus of the cell, but DJ-1 is recruited to mitochondria under oxidative conditions [11], [21]. More recently, several groups reported that loss of DJ-1 leads to mitochondrial abnormalities [22], [23], [24], [25]. However, it is less clear how DJ-1 and oxidative stress are involved in the regulation of mitochondrial function.

In the current study, we used primary mouse embryonic fibroblasts (MEFs) derived from *DJ-1*-deficient and wild-type mice to study how DJ-1 may be involved in the regulation of mitochondrial function. We found that loss of DJ-1 causes decreased mitochondrial transmembrane potential and increased mitochondrial permeability transition pore (mPTP) opening, though mitochondrial respiration is unaffected. While mitochondrial calcium levels are normal, ROS production is increased in mitochondria of *DJ-1*−/− MEFs. Furthermore, the decreased mitochondrial transmembrane potential and the increased mPTP opening in *DJ-1*−/− MEFs are restored by treatment of antioxidant molecules, suggesting that increased ROS production may underlie the mitochondrial defects. Together these findings highlight the antioxidant role of DJ-1 in the regulation of mitochondrial function.

## Materials and Methods

### Ethics Statement

The animal protocol used in the study was approved by the Harvard Center for Animal Resources and Comparative Medicine.

### Mouse Embryonic Fibroblast Preparation

MEFs (+/+ and −/−) were derived from embryos resulting from breeding heterozygous *DJ-1*+/− mice, which were described previously in [26]. Specifically, at embryonic day 14.5 embryos were obtained by cesarean section, and the head and inner organs were removed. Each embryo was individually minced with scissors, incubated twice with 1 ml of 1× trypsin-EDTA (Gibco, Life Technologies, Grand Island, NY, USA) for 10 min at 37°C and dissociated by pipetting vigorously. MEFs derived from embryos were grown and maintained in Dulbecco’s modified Eagle’s medium (DMEM, Gibco) supplemented with 10% fetal bovine serum (HyClone, Logan, UT, USA) at 37°C in a humidified incubator with 95% air and 5% CO_2_. After identification of *DJ-1*−/− and +/+ embryos by genotyping, MEFs from each embryo were expanded and frozen in DMEM containing 10% DMSO (Sigma, St Louis, MO, USA). The number of MEF cells used in each experiment and the number of embryos used to prepare the MEFs are specified in the figure legend.

### Mitochondrial Respiration Assay

Mitochondrial respiration was evaluated as O_2_ consumption in cell suspension using a Clark electrode (Rank Brothers Ltd, Cambridge, England). Cells were resuspended to a final density of 2×10^6^ cells/ml in respiration buffer (0.137 M NaCl, 5 mM KCl, 0.7 mM NaH_2_PO_4_, 25 mM Tris, pH 7.4 at 25°C). The oxygen electrode was calibrated with air-saturated water, assuming 406 nmol of oxygen per ml. Endogenous respiration activity was measured after addition of 10 mM glucose to the recording chamber. For substrate-specific respiration, plasma membranes were permeabilized by addition of digitonin at a final concentration of 0.01% (Sigma). Cells were then supplemented with substrates for either complex I (10 mM glutamate/malate, Sigma), II (10 mM succinate, Sigma) or III (1 mM TMPD/1 mM ascorbate, Sigma). After addition of 1 mM adenosine diphosphate (ADP, Sigma) to the recording chamber, State 3 respiration activity was measured. ADP independent respiration activity (State 4) was monitored after addition of oligomycin (2 µg/ml, Sigma). The Seahorse XF24 extracellular flux analyzer (Seahorse Bioscience, North Billerica, MA, USA) was also used to assess the oxygen consumption rate (OCRs; as indicator of mitochondrial respiration). 40,000 fibroblasts were transferred to each well in Seahorse XF24 plates. OCR was measured the following day on the XF24 flux analyzer. Three replicate OCR measurements were obtained at baseline and following injection of oligomycin (1 µM), carbonylcyanide-p-trifluoromethoxyphenylhydrazone (FCCP, 4 µM, Sigma) and rotenone (100 nM, Sigma). The value of the basal respiration, mitochondrial proton leak, maximal respiration, and nonmitochondrial respiration was determined as described in the Seahorse Operator’s Manual. Results were normalized to protein concentration. The metabolic capacity of mitochondria in the *DJ-1−/−* and control cortex was measured similarly to what was previously described in [27]. *DJ-1−/−* and littermate *DJ-1+/+* mice were used at the ages of 3 and 24–26 months.

### Measurement of the Specific Enzymatic Activities of the Individual Complexes in the ETS

All assays were performed on mitochondria isolated from MEFs according to a previously established method [28]. All spectrophotometric measures were conducted on a Benchmark plus 96 well plate reader (Perkin Elmer, Waltham, MA, USA). Complex I (NADH: ubiquinone oxidoreductase) activity was determined by adding 100 µl of assay buffer (35 mM NaH_2_PO_4_ pH 7.2, 5 mM MgCl_2_, 0.25% BSA, 2 mM KCN, 1 µM antimycin, 97.5 µM ubiquinone-1, 0.13 mM NADH, Sigma) to 5 µg of mitochondrial proteins. Only the rotenone sensitive activity was considered, and activities were monitored following the oxidation of NADH at 340 nm (OD 6220 M^−1^.cm^−1^). Complex II (succinate dehydrogenase) activity was determined by adding 100 µl of assay buffer (25 mM KH_2_PO_4_, 5 mM MgCl_2_, pH 7.2, 20 mM succinate, 50 µM DCPIP, 0.25% BSA, 2 mM KCN, 1 µM antimycin, Sigma) to 5 µg of mitochondrial proteins. Enzymatic activity was monitored spectrophotometrically by the reduction of dichloroindophenol/phenazine ethosulfate (DCPIP/PES) at 600 nm (OD 19100 M^−1^.cm^−1^). Complex IV (Cytochrome C oxidase) activity was determined following the oxidation of reduced Cytochrome C at 550 nm (OD 18500 M^−1^.cm^−1^) by adding 100 µl of assay buffer (30 mM KH_2_PO_4_ pH 7.4, 1 mM EDTA, 56 µM Cytochrome C, Sigma) to 5 µg mitochondrial proteins. Measurement of Adenosine-5′-triphosphate (ATP) concentration was performed using the Enliten ATP Assay Kit from Promega according to manufacturer’s instruction (Promega, Madison, WI, USA).

### Western Analysis

MEFs were collected in PBS-EDTA (5 mM) and centrifuged. The cells were sonicated in the lysis buffer (150 mM NaCl, 10 mM Tris, pH 7.4, 5 mM EDTA, 0.5% Triton X-100, 0.5% sodium dodecylsulfate, Sigma) containing protease and phosphatase inhibitors (Sigma). Fifty µg of protein per lane was resolved on 4–12% Bis-Tris gels (Invitrogen, Life Technologies), transferred to nitrocellulose membrane, blocked with blocking solution (LI-COR Bioscience, Lincoln, NE, USA), and incubated at 4°C overnight with total OXPHOS rodent WB antibody cocktail (mouse, MitoSciences, Eugene, OR, USA), primary antibodies against DJ-1 (rabbit, Abcam, Cambridge, MA, USA), superoxide dismutase 1 (SOD1, rabbit, Acris Antibodies, San Diego, CA, USA), superoxide dismutase 2 (SOD2, rabbit, Enzo Life Sciences, Farmingdale, NY, USA), catalase (rabbit, Calbiochem, Billerica, MA, USA), Glucose-6-phosphate dehydrogenase (G6PDH, rabbit, Sigma) and α-tubulin (mouse, Sigma). After these incubation membranes were washed three times in PBS containing 0.1% Tween and incubated with IRDye 680 or IRDye 800CW -conjugated secondary antibodies (LI-COR). Two hours later membranes were washed again, three times in 1× PBS containing 0.1% Tween, following by a final wash in 1× PBS then analyzed using an Odyssey Infrared Imaging System (LI-COR).

### Mitochondrial Transmembrane Potential

Mitochondrial transmembrane potential was measured with the non-quenching tetramethylrhodamine methyl ester (TMRM) fluorescence method. MEFs were cultured in the presence or absence of glutathione (10 mM, 24 hr, Sigma), N-Acetyl-Cystein (NAC, 20 mM, 24 hr, Sigma), H_2_O_2_ (500 µM, 3 hr, Sigma) or pyocyanin (100 µM, 24 hr, Sigma). Trypsinized cells were incubated in DMEM with 50 nM TMRM (Molecular Probes, Life Technologies) for 45 min at 37°C in the dark and washed in 1× HBSS. The TMRM signal was analyzed using the FACSCalibur flow cytometer (BD Biosciences) at the excitation wavelength of 585 nm (FL-2). For each experiment, TMRM fluorescence from 30,000 cells was acquired using the FACSCalibur flow cytometer and the median value was obtained using the FlowJo software (TreeStar Inc., Ashland, OR, USA). Gating was set the same way in all measurements. To examine the effect of antioxidant or oxidant treatment on mitochondrial transmembrane potential, MEFs cultured on glass bottom culture dishes were preincubated in the presence or absence of glutathione (10 mM, 24 hr), NAC (20 mM, 24 hr), H_2_O_2_ (500 µM, 3 hr) or pyocyanin (100 µM, 24 hr). Cells were loaded with 50 nM TMRM and 200 nM Mitotracker Green (Molecular Probes) in 1× HBSS for 20 min at 37°C and washed in 1× HBSS. Live images of cell were captured sequentially for TMRM fluorescence (excitation: 559 nm, emission range: 575–675 nm) and Mitotracker Green (excitation: 473 nm, emission range: 490–540 nm) using an Olympus FluoView FV1000 confocal microscope (Olympus Imaging America Inc, Center Valley, PA, USA). The quantification of the fluorescence was analyzed using the ImageJ software.

### Mitochondrial Permeability Transition Pore Opening

Mitochondrial permeability transition pore (mPTP) opening was assessed using the calcein-cobalt assay [29]. MEFs were cultured in the presence or absence of glutathione (10 mM, 24 hr), NAC (20 mM, 24 hr), H_2_O_2_ (500 µM, 3 hr) or pyocyanin (100 µM, 24 hr). Trypsinized cells were incubated in DMEM with calcein-AM (1 µM, Molecular Probes) at 37°C in the dark. After 30 min, CoCl_2_ (1 mM, Sigma) was added and the cells were incubated for another 10 min at 37°C in the dark. The fluorescence signal of mitochondria-trapped calcein was analyzed using a FACSCalibur flow cytometer at the excitation wavelength of 530 nm (FL-1). For each experiment, calcein fluorescence from 30,000 cells was acquired using FACSCalibur flow cytometer and the median value analysis was obtained using the FlowJo software. To determine the effect of antioxidant or oxidant treatment on mPTP, MEFs were cultured on glass bottom culture dishes with or without glutathione (10 mM, 24 hr), NAC (20 mM, 24 hr), H_2_O_2_ (500 µM, 3 hr) or pyocyanin (100 µM, 24 hr), then loaded with calcein-AM (1 µM) in the presence or absence of CoCl_2_ (1 mM), and Mitotracker Red (150 nM, Molecular Probes) in 1× HBSS for 20 min at 37°C and washed in 1× HBSS. Images of live cells were captured sequentially for calcein fluorescence (excitation: 473 nm, emission range: 490–540 nm) and Mitotracker Red (excitation: 559 nm, emission range: 575–675 nm) using an Olympus FluoView FV1000 confocal microscope. The quantification of the fluorescence was analyzed using the ImageJ software.

### Reactive Oxygen Species Measurement

ROS production was measured using 3 different specific dyes. Intracellular H_2_O_2_ production was determined by measuring the fluorescence intensity of Amplex Red dye (Molecular Probes) in isolated mitochondria using the mitochondrial isolation kit from Sigma following the manufacturer’s instruction or in intact cells. Intracellular superoxidase anion and mitochondrial superoxide production were measured in intact cells using Dihydroethidium (DHEt, Molecular Probes) and Mitotracker CM-H_2_XROS (Molecular Probes), respectively. For each dye, MEFs or isolated mitochondria were resuspended in 1× HBSS and loaded with 100 µl of Amplex Red Buffer (10 µM Amplex Red, 10 mM succinate, 0.2 units/ml Horse Radish Peroxidase, Sigma), DHEt (2 µM in 1× HBSS) or CM-H_2_XROS (2 µM in 1× HBSS) in 96 well plates. The time course of changes of fluorescence spectra was measured using the Synergy HT (Bioteck, Winooski, VT) plate reader. Amplex Red and DHEt were excited at 530±12.5 nm, and their emission was measured at 590±17.5 nm, whereas CM-H_2_XROS Mitotracker was excited at 560±10 nm, and its emission was measured at 620±20 nm. For live cell imaging of ROS production, MEFs in glass bottom dishes were loaded with Mitotracker Green (200 nM) and Amplex Red (2.5 µM), DHEt (2.5 µM) or MitoSOX Red (2.5 µM, Molecular Probes) in 1× HBSS for 30 min at 37°C and then washed 3 times with 1× HBSS. Images were captured sequentially for Amplex Red, DHEt and MitoSOX fluorescence (excitation: 559 nm, emission range: 575–675 nm) and Mitotracker Green (excitation: 473 nm, emission range: 490–540 nm) using an Olympus FluoView FV1000 confocal microscope. The quantification of the fluorescence was analyzed using the ImageJ software.

### Calcium Imaging

The FCCP releasable pool of intracellular calcium was measured by adapting a previously described method [30]. Briefly, MEFs were loaded with Fura-2 AM (5 µM) (Molecular probes) in HCSS buffer (120 mM NaCl, 5.4 mM KCl, 0.8 mM MgCl_2_, 2 mM CaCl_2_, 15 mM glucose and 20 mM HEPES, pH 7.3) for 45 min at 37°C and imaged using a Leica DMI6000 microscope (Leica Microsystems GmbH, Wetzlar, Germany). After 10 sec of recording, cells were incubated for 10 more sec in HCSS-Ca^2+^ deficient buffer containing EGTA (5 mM) and then treated with FCCP (1 µM) in HCSS- Ca^2+^ deficient buffer using an 8-channel gravity perfusion system (ALA Scientific Instrument, Farmingdale, NY, USA). Imaging processing and data analysis were performed using the LAS AF software (Leica).

### Statistical Analysis

Pooled results are expressed as means ± SEM. Statistical analysis was performed with the GraphPad Prism software (GraphPad Software, Inc., San Diego, CA, USA) using the non paired Student *t*-test for pairwise comparisons or using the ANOVA Newmann-Keuls multiple comparison test for one-way analysis of variance.

## Results

### Normal Mitochondrial Respiration in Primary *DJ-1−/−* MEFs and the *DJ-1−/−* Cerebral Cortex

**Figure 1:**
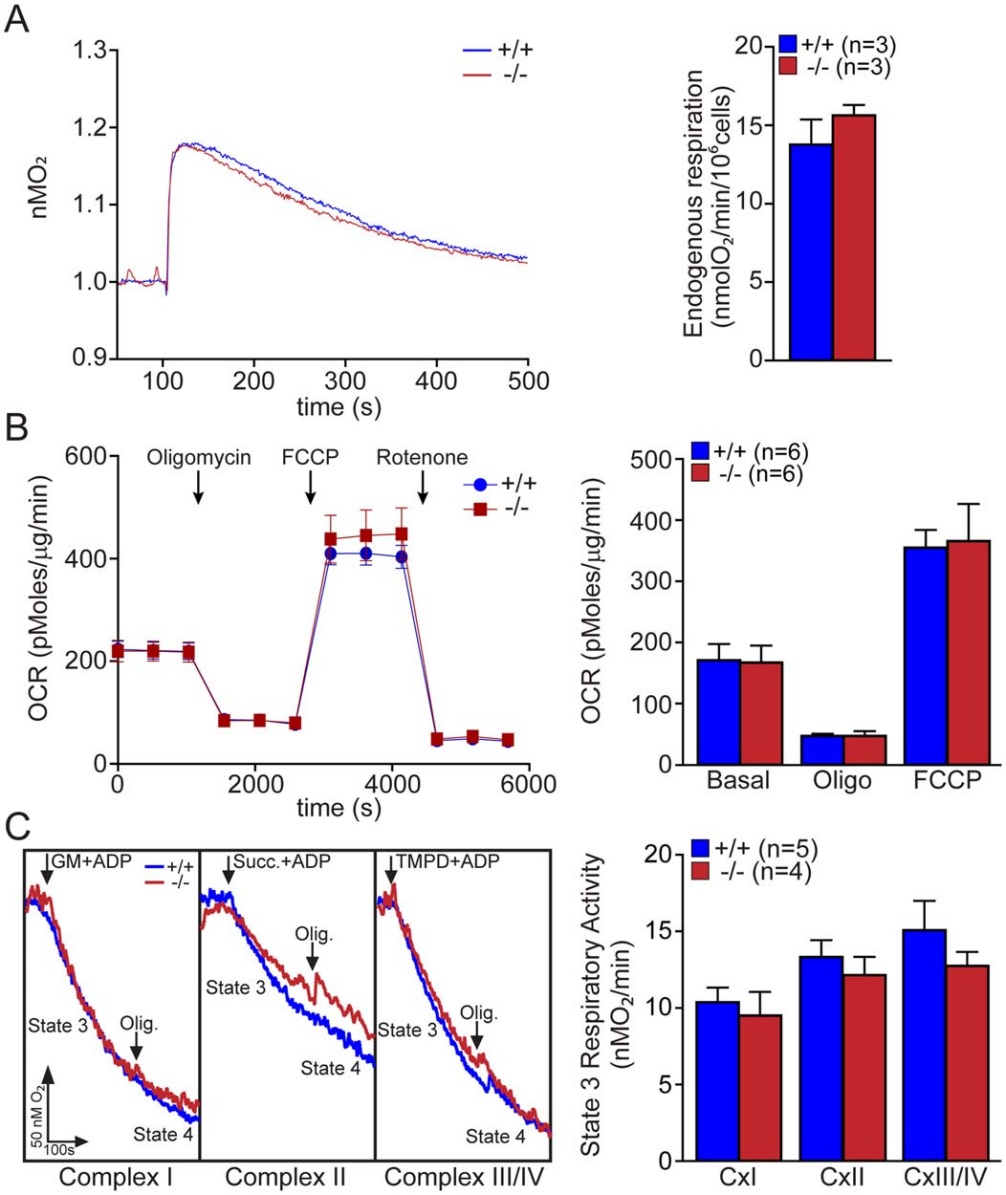
Normal mitochondrial respiration in intact mitochondria of *DJ-1−/−* MEFs. (**A**) Endogenous respiratory activity in *DJ-1*−/− and +/+ MEFs. Representative oxygraphs of *DJ-1*−/− and +/+ MEFs energized with glucose (10 mM) are shown on the left. The bar graph on the right shows oxygen consumption, which represents the endogenous respiratory activity in *DJ-1*−/− and +/+ MEFs. The data were obtained from three independent experiments using primary MEFs obtained from 3 individual embryos per genotype. (**B**) Oxygen consumption rate (OCR) profile in *DJ-1*−/− and +/+ MEFs. OCR profile expressed as pMolesO_2_/min in control and *DJ-1*−/− cells are shown on the left. Arrows indicate the time of addition of oligomycin (Oligo, 1 µM), FCCP (4 µM) and rotenone (100 nM). The bar graph on the right shows OCRs normalized to protein concentration after subtraction of rotenone insensitive OCR (nonmitochondrial respiration), under basal condition, after addition of oligomycin (Oligo, 1 µM, proton leak) or FCCP (4 µM, maximal respiration). The data were obtained from three independent experiments using primary MEFs obtained from 6 individual embryos. (**C**) Energized respiration in *DJ-1*−/− and +/+ MEFs. Representative traces of respiration rates in the mitochondria in *DJ-1*−/− and +/+ MEFs are shown on the left. Arrows indicate the application of substrates (complex I: 10 mM glutamate/malate (GM), complex II: succinate (Succ, 10 mM), complex III/IV: 1 mM TMPD/1 mM ascorbate (TMPD)) in the presence of ADP (1 mM) and oligomycin (oligo). The bar graphs on the right show state 3 respiratory activity for complex I, II and III/IV in *DJ-1*−/− and +/+ MEFs permeabilized with digitonin. The number shown in the panel indicates the number of embryos used to derive primary MEFs per genotype, and the data were obtained from three independent experiments. All data are expressed as the mean ± S.E.

A recent report showed that mitochondrial respiration is impaired in immortalized *DJ-1*−/− MEFs [22]. To investigate how loss of DJ-1 affects mitochondrial respiration, we established primary MEFs from *DJ-1*−/− and wild-type embryos and then assessed mitochondrial respiratory activity in these MEFs. We first measured endogenous respiratory activity of primary MEFs energized with glucose (10 mM) and did not find any difference in the endogenous respiration between *DJ-1*−/− and +/+ MEFs (Fig. 1A). Using Seahorse XF24 we also measured basal and maximal respiration, and still did not find any genotypic differences in basal and maximal respiration between *DJ-1*−/− and +/+ MEFs (Fig. 1B). The maximal respiration was determined after sequential addition of complex V inhibitor oligomycin and mitochondrial uncoupler FCCP minus the nonmitochondrial respiration evaluated by rotenone, an inhibitor of complex I activity [31].

We then examined the metabolic capacity of mitochondria in digitonin-permeabilized *DJ-1*−/− and +/+ MEFs using substrates specific for each complex: complex I (10 mM glutamate/malate), complex II (10 mM succinate), and complex III/IV (1 mM TMPD/1 mM ascorbate). We measured state 3, which represents the maximum respiration rate in the presence of saturating ADP (1 mM) and state 4, which represents oxygen consumption by leakage of protons through the inner membrane after ADP exhaustion. The use of digitonin (0.01% final concentration) allows the direct delivery of substrates to mitochondria by permeabilizing the plasma membrane without affecting mitochondrial integrity. Representative traces for each substrate-mediated respiration in *DJ-1*−/− and +/+ MEFs are shown in Fig. 1C. There are no significant differences between *DJ-1*−/− and +/+ MEFs in state 3 (Fig. 1C) and state 4 (data not shown) respiratory activities for complex I, complex II, and complex III/IV as well as in the state 3/state 4 respiratory control ratio (data not shown).

**Figure 2:**
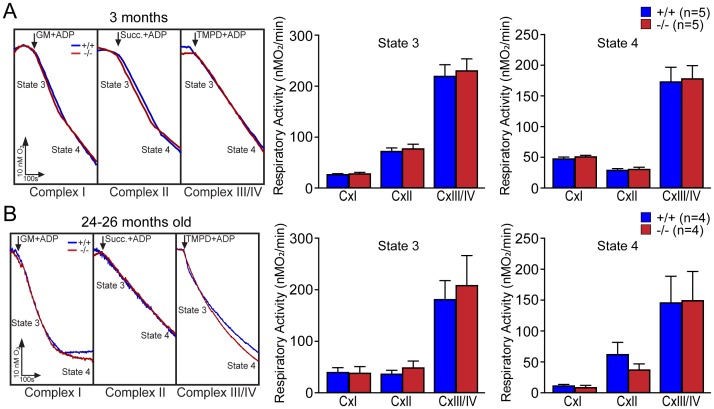
Normal mitochondrial respiration in isolated mitochondria from the cortex of *DJ-1*−/− mice. (**A**) Energized respiration in mitochondria isolated from the cerebral cortex of *DJ-1*−/− and +/+ mice at 3 months of age. Representative traces of respiration rates in mitochondria isolated from the cortex of *DJ-1*−/− and littermate control mice are shown on the left. Arrows indicate the time of the application of substrates (complex I: 10 mM glutamate/malate (GM), complex II: succinate (Succ, 10 mM), complex III/IV: 1 mM TMPD/1 mM ascorbate (TMPD)) in the presence of ADP (1 mM). The bar graphs on the right show state 3 and state 4 respiratory activities for complex I, II and III/IV in isolated mitochondria from the cortex of *DJ-1*−/− and +/+ mice at the age of 3 months. (**B**) Energized respiration in mitochondria isolated from the cortex of 24–26 months old *DJ-1*−/− and +/+ mice. Representative traces of respiration rates in mitochondria isolated from the cortex of 24–26 months old *DJ-1*−/− and control mice are shown on the left. Arrows indicate the time of the application of substrates (complex I: 10 mM glutamate/malate (GM), complex II: succinate (Succ, 10 mM), complex III/IV: 1 mM TMPD/1 mM ascorbate (TMPD)) in the presence of ADP (1 mM). The bar graphs on the right show state 3 and state 4 respiratory activities for complex I, II and III/IV in isolated mitochondria from the cortex of *DJ-1*−/− and control mice at 24–26 months of age. The number shown in the panel indicates the number of mice used in the study. All data are expressed as the mean ± S.E.

Using the same technique we also assessed the metabolic capacity of mitochondrial crude preparation isolated from the cerebral cortex of *DJ-1*−/− and +/+ littermate mice at the ages of 3 and 24–26 months. We measured state 3 and state 4 activities for each complex using specific substrates. Representative traces for each substrate-mediated respiration of isolated cortical mitochondria from *DJ-1*−/− and +/+ mice are shown in Fig. 2A (3 months) and 2B (24–26 months). There are no significant differences in state 3 and state 4 respiration for complex I, complex II, or complex III/IV in isolated mitochondria between *DJ-1*−/− and +/+ mice at 3 months of age (Fig. 2A) and at 24–26 months of age (Fig. 2C). Together these results show that loss of DJ-1 does not result in impairment of mitochondrial respiration in intact mitochondria from primary MEFs and in isolated mitochondria from the cerebral cortex.

### Decreased ATP Levels but Normal Activities of Enzymes in the Mitochondrial ETS in *DJ-1−/−* MEFs

**Figure 3:**
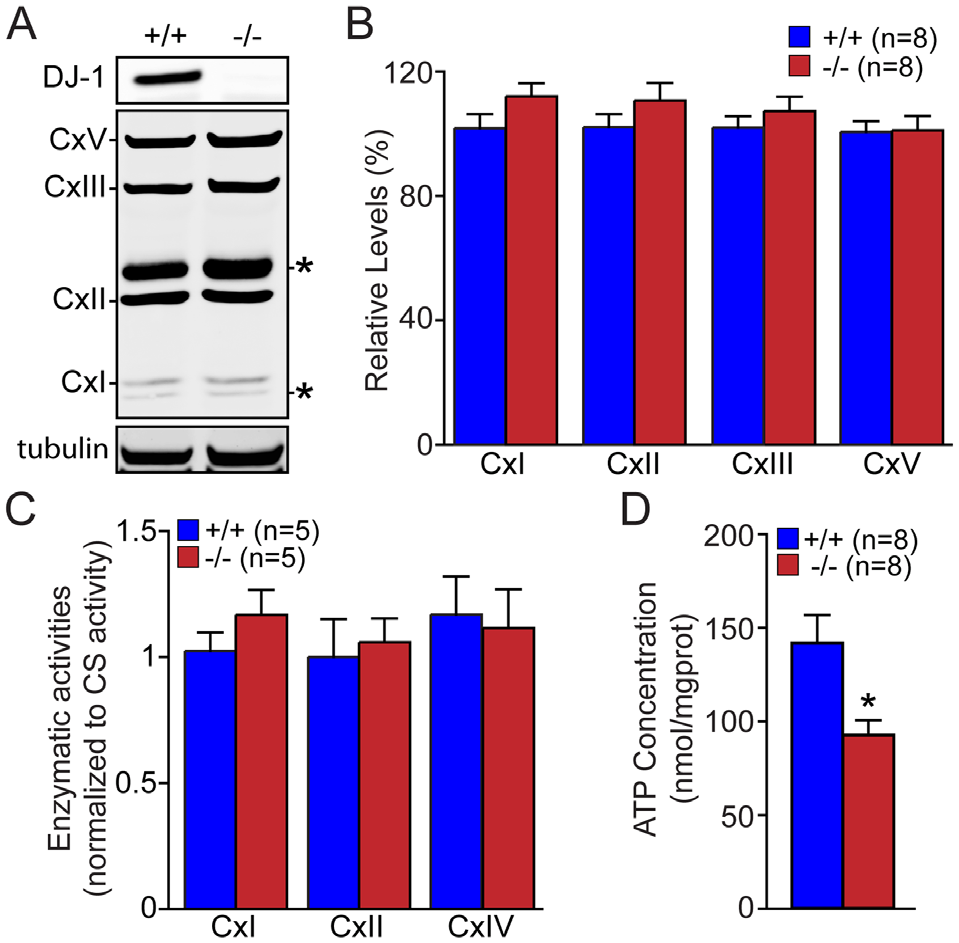
Decreased ATP concentration in *DJ-1*−/− MEFs. (**A, B**) Western analysis of each subunit in the oxidative phosphorylation (OXPHOS) complex in *DJ-1*−/− and +/+ MEFs. (**A**) Representative western blot showing relative expression of each subunit. Tubulin was used as loading control. Non-specific bands are marked by asterisk. (**B**) The bar graph shows the quantification and normalization of the expression level of each subunit using tubulin as loading control. (**C**) Enzymatic activities of complexes I, II and IV of the mitochondrial electron transport system, as measured by spectrophotometric assays and after normalization to citrate synthase activity (CS). (**D**) The bar graph shows decreased ATP concentrations in *DJ-1*−/− MEFs compared to control cells. The number shown in the panel indicates the number of embryos used to derive primary MEFs per genotype, and the data were obtained from three independent experiments. All data are expressed as mean ± SEM. *p<0.05.

We then measured the enzymatic activities and expression levels of all individual complexes composing the electron transport system (ETS) in *DJ-1*−/− and +/+ MEFs (Fig. 3A–C). There is no significant difference in the expression level of each complex protein in respiratory chain between *DJ-1*−/− and +/+ MEFs (Fig. 3A and 3B). Furthermore, we measured the specific enzymatic activity of the individual complex of the respiratory chain, complex I (NADH-ubiquinone reductase activity), complex II (succinate-ubiquinone reductase activity) and complex IV (Cytochrome oxidase activity) using isolated mitochondria from *DJ-1*−/− and +/+ MEFs (Fig. 3C). After normalization to citrate synthase activity, enzymatic activities of all complexes composing the ETS appear normal in *DJ-1*−/− MEFs. We then measured the level of ATP in *DJ-1*−/− and +/+ MEFs using a luciferin/luciferase assay that provides a direct quantification of ATP concentration [32], [33]. Interestingly, lack of DJ-1 leads to a decrease of ATP concentration in *DJ-1*−/− MEFs (p<0.05, n = 8, Fig. 3D). Thus, loss of DJ-1 does not affect levels of mitochondrial complexes and activities but does cause reduction of ATP concentration.

### Decreased Mitochondrial Transmembrane Potential in *DJ-1−/−* MEFs

**Figure 4:**
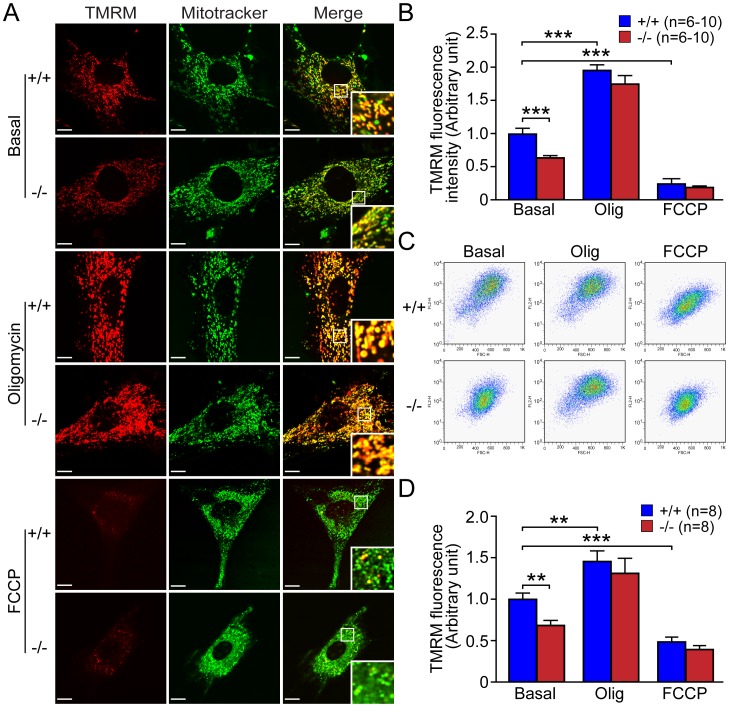
Reduced mitochondrial membrane potential (ΔΨ_m_) in *DJ-1*−/− MEFs. (**A, B**) Confocal microscopic analysis. (**A**) Representative confocal microscopic images of *DJ-1*−/− and +/+ MEFs after staining with TMRM (50 nM, red) and Mitotracker Green (200 nM) in the presence or absence of oligomycin (Olig, 1 µM) or FCCP (10 µM). The intensity of TMRM reflects the level of ΔΨ_m_, whereas the intensity of Mitotracker Green is not affected by transmembrane potential. Insets in panels indicate higher power views of the boxed area. Scale bar: 10 µm. (**B**) The bar graph shows quantification of TMRM signal in *DJ-1*−/− and +/+ MEFs in the presence or absence of oligomycin or FCCP. The TMRM signal is reduced in *DJ-1*−/− cells relative to wild-type cells, whereas the TMRM signal is increased or decreased in both *DJ-1*−/− and +/+ cells following oligomycin or FCCP treatment, respectively. The number shown in the panel indicates the number of cells quantified per genotype in the study. (**C, D**) FACS analysis. (**C**) Representative flow cytometric dot plots show the intensity of TMRM signal in *DJ-1*−/− and +/+ MEFs following incubation with TMRM (50 nM) in the presence or absence of oligomycin (1 µM) or FCCP (10 µM). (**D**) The bar graph shows quantification of TMRM signal measured by FACS analysis in *DJ-1*−/− and +/+ MEFs. The number shown in the panel indicates the number of embryos used to derive primary MEFs per genotype, and the data were obtained from five independent experiments. All data are expressed as mean ± SEM. **p<0.01, ***p<0.001.

In the absence of enzymatic defects of the ETS complexes but decreased ATP levels, we turned our attention to mitochondrial transmembrane potential (Δ Ψ_m_), the electrochemical force that modulates the kinetics of proton reentry to the matrix through ATP-synthase. Using two different methods, live cell imaging (Fig. 4A and 4B) and flow cytometry (Fig. 4C and 4D), we measured mitochondrial transmembrane potential in *DJ-1*−/− and +/+ MEFs stained with TMRM (50 nM). TMRM is a cationic fluorescent dye that accumulates inside mitochondrial matrix according to membrane potential [34]. We found that mitochondrial transmembrane potential measured by TMRM fluorescence signal using both microscopic (p<0.001, n = 6–10) and FACS (p<0.01, n = 8) analyses is reduced in *DJ-1*−/− MEFs (Fig. 4). To ensure equal dye loading and non-quenching mode, we compared TMRM fluorescence in *DJ-1*−/− and control MEFs following administration of oligomycin and FCCP. Oligomycin, an inhibitor of ATP synthase, induces hyperpolarization of mitochondria, whereas FCCP dissipates transmembrane potential. As expected, oligomycin induced marked increases of TMRM fluorescence in both genotypic groups (p<0.01), while FCCP treatment caused drastic decreases of TMRM fluorescence (p<0.01), and these treatments eliminated the genotypic difference in TMRM fluorescence between *DJ-1*−/− and +/+ MEFs (Fig. 4). Thus, loss of DJ-1 results in reduction of mitochondrial transmembrane potential.

### Increased Mitochondrial Permeability Transition Pore Opening in *DJ-1−/−* MEFs

**Figure 5:**
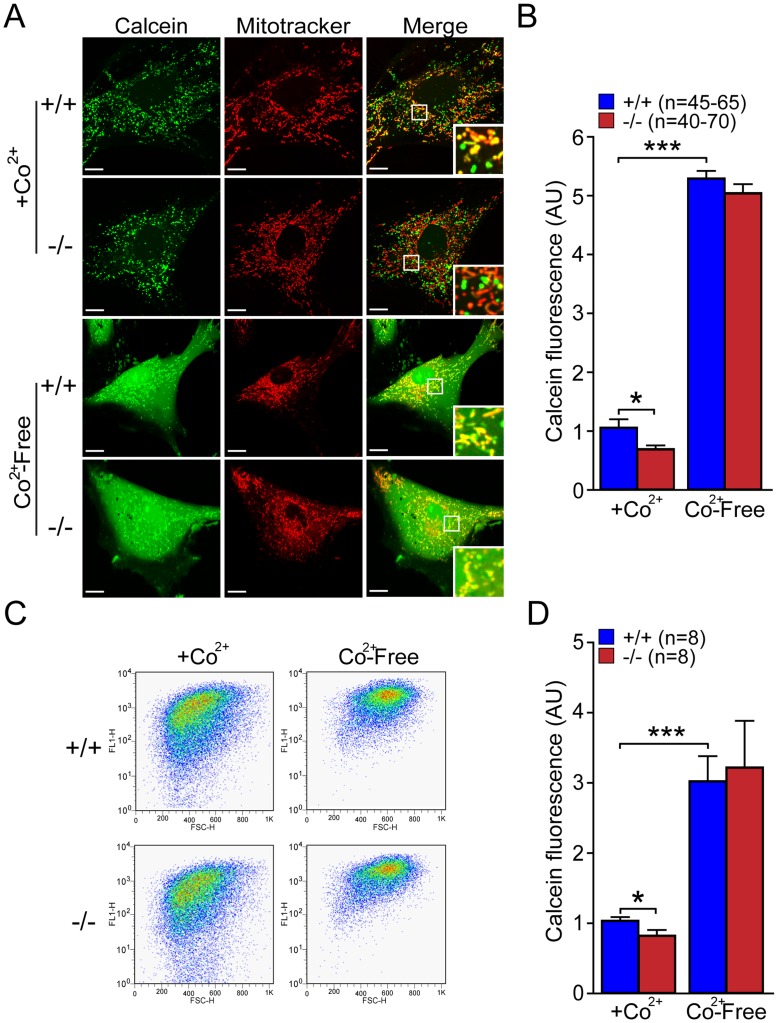
Increased opening of mitochondrial permeability transition pore in *DJ-1*−/− cells. (**A, B**) Confocal microscopy analysis. (**A**) Representative confocal microscopic images of *DJ-1*−/− and +/+ MEFs after incubation with calcein-AM (1 µM, green) and Mitotracker Red (150 nM) in the presence or absence of Co^2+^ (1 mM), which quenches calcein fluorescence (green) outside of mitochondria. Mitotracker Red confirms the localization of calcein fluorescence in mitochondria. Insets indicate higher power views of the boxed area in the panel. The calcein fluorescence in mitochondria is lower in *DJ-1*−/− cells in the presence of Co^2+^. In the absence of Co^2+^, calcein fluorescent signals are very intense and are present in the entire cell, and there are no genotypic differences. Scale bar: 10 µm. (**B**) The bar graph shows quantification of calcein fluorescence in *DJ-1*−/− and +/+ cells in the presence or absence of Co^2+^. The number shown in the panel indicates the number of cells quantified per genotype in the study. (**C, D**) FACS analysis. (**C**) Representative flow cytometric dot plots show the intensity of calcein signal in *DJ-1*−/− and +/+ MEFs following incubation with calcein-AM (1 µM) in the presence or absence of Co^2+^ (1 mM). (**D**) The bar graph of calcein signal measured by FACS analysis shows reduced calcein signal in *DJ-1*−/− MEFs in the presence of Co^2+^. The number shown in the panel indicates the number of embryos used to derive primary MEFs per genotype, and the data were obtained from five independent experiments. All data are expressed as mean ± SEM. *p<0.05, ***p<0.001.

To investigate the mechanism underlying the reduction of mitochondrial transmembrane potential, we evaluated the opening of the mitochondrial permeability transition pore, which allows the diffusion of small ions across the mitochondrial inner membrane [35]. We measured mPTP opening in *DJ-1*−/− and +/+ MEFs under basal conditions using the CoCl_2_-calcein fluorescence-quenching assay [36]. We loaded the cells with calcein-AM, a membrane permeable fluorophore that is able to diffuse freely in all subcellular compartments including mitochondria. Calcein, which is hydrophilic, is then trapped in all subcellular compartments after the cleavage of its acetoxymethyl (AM) group by ubiquitous intracellular esterase. Cells were then incubated with the divalent cobalt cation (Co^2+^), which is able to quench calcein fluorescence in all subcellular compartments except the mitochondrial matrix, as the inner mitochondrial membrane is the only intracellular membrane that is Co^2+^-impermeable [36], [37]. When mPTP is open, cobalt is able to enter into mitochondria and quench mitochondrial calcein fluorescence [38]. Using two different methods, microscopic (Fig. 5A and 5B) and flow cytometric (Fig. 5C and 5D) analyses, we measured calcein fluorescence in *DJ-1*−/− and +/+ MEFs. We found decreases of calcein fluorescence in *DJ-1*−/− MEFs compared to the control using both microscopic (p<0.05, n = 40–70) and FACS (p<0.05, n = 8) analyses. We also confirmed equal loading of the dye between *DJ-1*−/− and +/+ MEFs by measuring calcein fluorescence in the absence of Co^2+^. As expected, we saw drastic increases of calcein fluorescence in the absence of Co^2+^ (p<0.001) and there is no genotypic difference between *DJ-1*−/− and +/+ MEFs in calcein fluorescence (Fig. 5C and 5D).

### Normal Mitochondrial Calcium Concentration in *DJ-1−/−* MEFs

**Figure 6:**
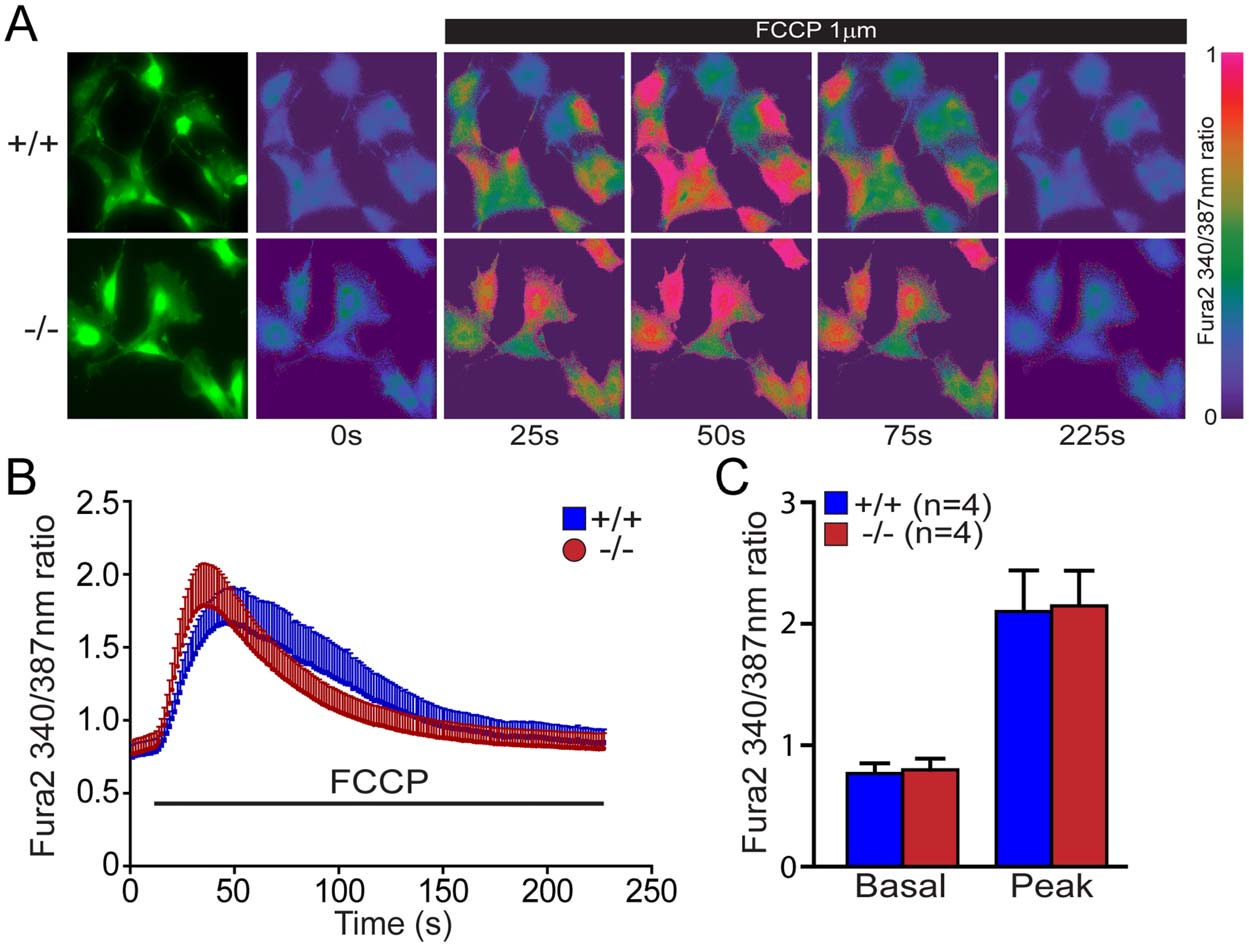
Normal levels of mitochondrial calcium in *DJ-1*−/− cells. (**A**) Representative Fura-2 images of Ca^2+^ responses following FCCP treatment in *DJ-1*−/− and +/+ MEFs. Fura-2 ratios at 340/387 are shown at time points indicated. The green fluorescence images show the shape of the *DJ-1*−/− and +/+ MEFs. The pseudocolor calibration scale for 340/387 ratios is shown on the right. FCCP (1 µM) was added at t = 25 s. (**B**) Time course of cytosolic [Ca^2+^] rise following FCCP treatment in *DJ-1*−/− and +/+ MEFs. (**C**) The basal and the peak value of cytosolic calcium rise following FCCP are the same in *DJ-1*−/− and +/+ MEFs. The number shown in the panel indicates the number of embryos used to derive primary MEFs, and the data were obtained from three independent experiments. All data are expressed as mean ± SEM.

Because mPTP opening can be induced by an elevation of mitochondrial calcium levels [39], we then measured the size of mitochondrial calcium pool by quantifying the amount of calcium released from mitochondria to the cytosol following FCCP treatment. FCCP is a specific proton ionophore that dissipates proton gradient and allows cation to redistribute freely across membranes according to their concentration gradient. Following FCCP treatment, alteration of cytosolic calcium concentration was monitored with Fura-2, a ratiometric fluorescent dye that binds to free intracellular calcium [40]. Fura-2 is excited at wavelengths 340 nm and 380 nm, and the ratio of the emissions is directly correlated to the amount of intracellular calcium. Increases in Fura-2 signal following FCCP treatment are the same between *DJ-1*−/− and +/+ MEFs (Fig. 6). Thus, loss of DJ-1 does not seem to affect the size of mitochondrial calcium pool.

### Increased Levels of Oxidative Stress in *DJ-1−/−* Cells

**Figure 7:**
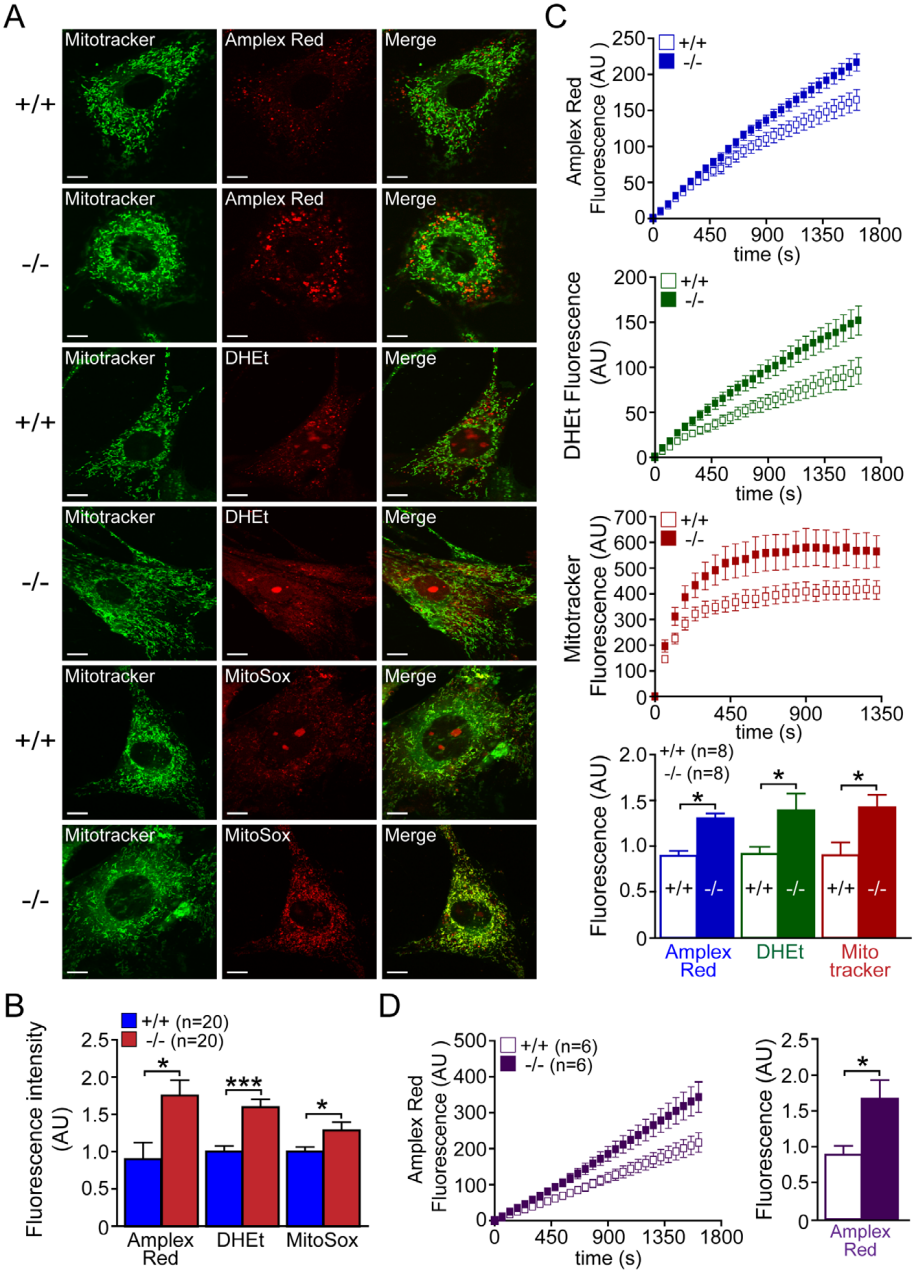
Increased reactive oxygen species (ROS) production in *DJ-1*−/− MEFs. (**A, B**) Confocal microscopy analysis of ROS concentration. (**A**) Representative confocal live cell images of *DJ-1*−/− and +/+ MEFs after incubation with Mitotracker Green (200 nM) and Amplex Red (2.5 µM), DHEt (2.5 µM) or MitoSOX Red (2.5 µM). Scale bar: 10 µm. (**B**) The bar graph shows the quantification and the increase of Amplex Red, DHEt or MitoSOX Red fluorescence in *DJ-1*−/− cells compared to control cells. The number shown in the panel indicates the number of cells quantified per genotype in the study. (**C**) Kinetics analysis of ROS production. The time course of the fluorescence changes in *DJ-1*−/− and +/+ MEFs labeled with Amplex Red (upper), DHEt (middle), or Mitotracker CM-H_2_XROS (lower) is shown. The bar graph at the bottom shows quantitative analysis of fluorescence changes, indicating significant increases of fluorescence signals of Amplex Red, DHEt and Mitotracker CM-H_2_XROS in *DJ-1*−/− MEFs. The number shown in the panel indicates the number of embryos used to derive primary MEFs per genotype, and the data were obtained from four independent experiments. (**D**) Kinetics of H_2_O_2_ production in isolated mitochondria measured by following Amplex Red fluorescence over time showing an increase of its production in *DJ-1*−/− MEFs. The number shown in the panel indicates the number of embryos used to derive primary MEFs per genotype, and the data were obtained from three independent experiments. All data are expressed as the mean ± S.E. *p<0.05, ***p<0.001.

**Figure 8:**
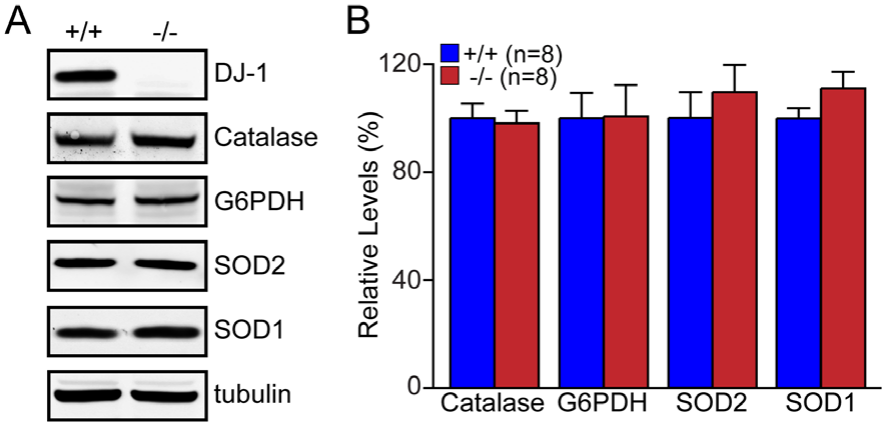
Normal levels of antioxidant proteins in *DJ-1*−/− MEFs. (**A**) Representative western blot showing expression levels of Catalase, G6PDH, SOD1 and SOD2. Tubulin was used as loading control. (**B**) The bar graph shows the quantification of the level of each protein normalized to tubulin. The number shown in the panel indicates the number of embryos used to derive primary MEFs per genotype, and the data were obtained from three independent experiments. All data are expressed as mean ± SEM.

While mitochondrial calcium appears unaffected in the absence of DJ-1, mPTP opening can also be influenced by elevated oxidative stress [35]. Furthermore, mitochondria are the major site where reactive oxygen species are produced in the cell [41], and several reports showed that DJ-1 can function as oxidative stress sensor and scavenger [11], [12], [14]. We therefore evaluated ROS production in whole cell and in mitochondrial fraction from *DJ-1*−/− and +/+ MEFs (Fig. 7). We first evaluated production of oxidative species using live cells loaded with Amplex Red, dihydroethidium (DHEt) or MitoSOX Red (Fig. 7A and 7B). The intensity of Amplex Red fluorescence is modulated by the amount of H_2_O_2_ produced in the cell [42], whereas the fluorescent intensity of DHEt and MitoSOX Red reflects production of intracellular superoxide (O_2_^·−^) and intramitochondrial superoxide, respectively [43], [44], [45]. We found that the intensities of all three fluorescent dyes are higher in *DJ-1*−/− MEFs (p<0.05, p<0.001, n = 20, Fig. 7A and 7B), indicating increases of ROS production in the absence of DJ-1. Mitotracker Green was used as control, since it is independent of oxidative conditions and membrane potential [46]. We then followed the increase of the fluorescence over time and found that increases of Amplex Red (p<0.05, n = 8), DHEt (p<0.05, n = 8) and Mitotracker CM-H_2_XROS (p<0.05, n = 8) fluorescence over time are higher in *DJ-1*−/− MEFs compared to control cells (Fig. 7C). Because H_2_O_2_ extrusion across the plasma membrane can be limiting, we also measured the rate of H_2_O_2_ produced using isolated mitochondria from *DJ-1*−/− and +/+ MEFs (Fig. 7D). We found that isolated mitochondria from *DJ-1*−/− MEFs produced more H_2_O_2_ than control MEFs (p<0.05, n = 6). We also performed positive control experiments using different amounts of H_2_O_2_ and found that the intensity of Amplex Red fluorescence directly correlated to the concentration of H_2_O_2_ in wild-type cells (data not shown). We further used pyocyanin to induce ROS in control cells, and found that the fluorescent intensity of Amplex Red, DHEt and Mitotracker CM-H_2_XROS is responsive to the induction of ROS production (data not shown). Since a recent report showed that DJ-1 influences expression levels of several antioxidative enzymes [47], [48], we measured levels of antioxidative enzymes. Western analysis showed no significant difference in the level of antioxidative enzymes including SOD1, SOD2, catalase, and G6PDH between *DJ-1*−/− and +/+ MEFs (Fig. 8A and 8B).

### Antioxidant Molecules Restore Reduced ΔΨ_m_ in *DJ-1−/−* MEFs and Oxidative Inducers Decrease ΔΨ_m_ in *DJ-1+/+* MEFs

**Figure 9:**
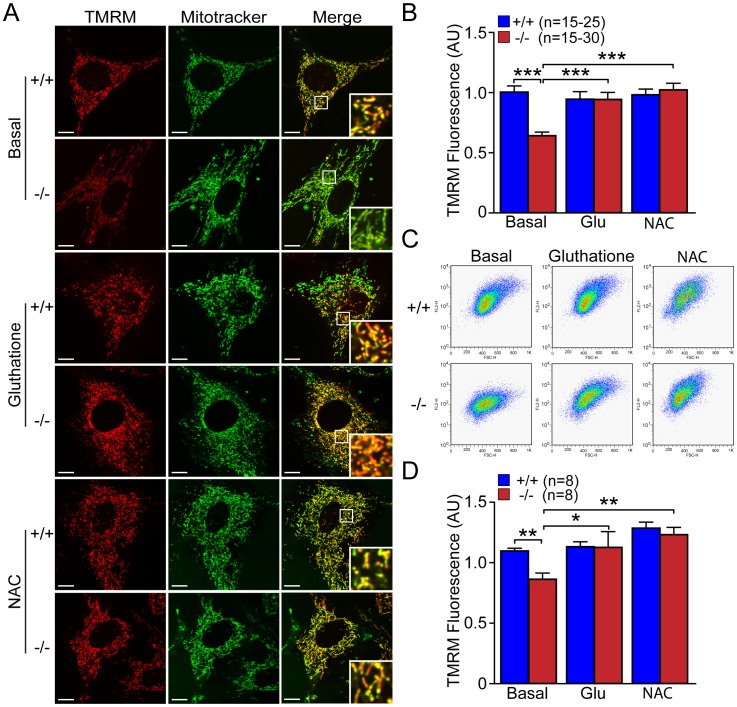
Antioxidant glutathione and NAC restore the reduced ΔΨ_m_ in *DJ-1*−/− MEFs. (**A, B**) Confocal microscopic analysis. (**A**) Representative confocal live cell images of *DJ-1*−/− and +/+ MEFs stained with TMRM (50 nM, red) and Mitotracker Green (200 nM) after incubation with or without glutathione (Glu, 10 mM, 24 hr) or NAC (20 mM, 24 hr). Insets show higher power views of the boxed area in the panel. Scale bar: 10 µm. (**B**) The bar graph shows quantification of TMRM signal in *DJ-1*−/− and +/+ MEFs after incubation with or without glutathione (Glu, 10 mM, 24 hr) or NAC (20 mM, 24 hr). The TMRM signal is reduced in *DJ-1*−/− cells under basal conditions, whereas the TMRM signal is increased in *DJ-1*−/− after incubation with antioxidants. The number shown in the panel indicates the number of cells quantified per genotype in the graph. (**C, D**) FACS analysis. (**C**) Representative flow cytometric dot plots show the intensity of TMRM signal in *DJ-1*−/− and +/+ MEFs following incubation with TMRM (50 nM) after incubation with or without glutathione (Glu, 10 mM, 24 hr) or NAC (20 mM, 24 hr). (**D**) The bar graph shows quantification of TMRM signal in *DJ-1*−/− and +/+ MEFs and the rescue of the decrease of the TMRM fluorescence in *DJ-1*−/− cells after incubation with antioxidant molecules. The number shown in the panel indicates the number of embryos used to derive primary MEFs per genotype, and the data were obtained from five independent experiments. All data are expressed as mean ± SEM. *p<0.05, **p<0.01, ***p<0.001.

To determine whether increased ROS production may underlie the reduced mitochondrial transmembrane potential in *DJ-1*−/− MEFs, we examined the effect of antioxidants or ROS inducers on mitochondrial transmembrane potential in *DJ-1*−/− and +/+ MEFs. Using both microscopic and flow cytometric analyses, we measured mitochondrial transmembrane potential in *DJ-1*−/− and +/+ MEFs after incubation with antioxidant molecules, such as glutathione and N-Acetyl-Cystein (NAC). We performed TMRM (50 nM) and Mitotracker Green (200 nM, as fluorescence intensity control) staining in *DJ-1*−/− and +/+ MEFs preincubated with or without glutathione (10 mM, 24 hr) or NAC (20 mM, 24 hr). Representative confocal live images (Fig. 9A) and quantification of TMRM staining (Fig. 9B) showed that TMRM signal intensity is increased in *DJ-1*−/− MEFs cultured in the presence of glutathione (p<0.001, n = 15–30) or NAC (p<0.001, n = 15–30), relative to basal conditions. Quantitative analysis of TMRM fluorescence following FACS showed significant increases of TMRM fluorescence in *DJ-1*−/− MEFs cultured with glutathione (p<0.05, n = 8) or NAC (p<0.01, n = 8), relative to basal conditions (Fig. 9C and 9D). Treatment of glutathione or NAC does not affect mitochondrial transmembrane potential in *DJ-1*+/+ MEFs (Fig. 9). These results show that the reduction in mitochondrial transmembrane potential in *DJ-1*−/− cells can be restored with antioxidant molecules.

**Figure 10 pone-0040501-g010:**
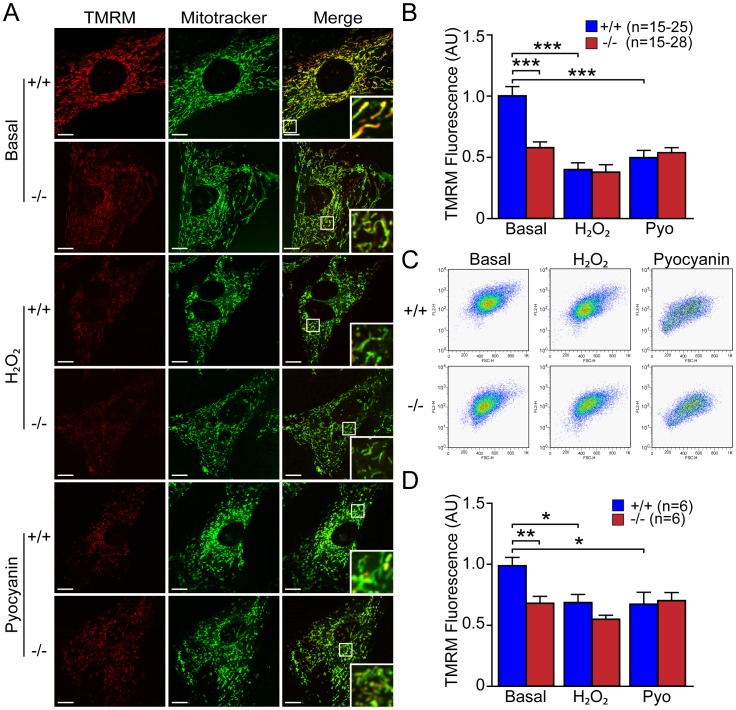
Increases of oxidative stress induce reduction of ΔΨ_m_ in *DJ-1*+/+ MEFs. (**A, B**) Confocal microscopic analysis. (**A**) Representative confocal live cell images of *DJ-1*−/− and +/+ MEFs stained with TMRM (50 nM, red) and Mitotracker Green (200 nM) after incubation with or without H_2_O_2_ (500 µM, 3 hr) or pyocyanin (100 µM, 24 hr). Insets show higher power views of the boxed area in the panel. Scale bar: 10 µm. (**B**) The bar graph shows quantification of TMRM signal in *DJ-1*−/− and +/+ MEFs after incubation with or without H_2_O_2_ (500 µM, 3 hr) or pyocyanin (100 µM, 24 hr). The TMRM signal is markedly reduced in *DJ-1*+/+ cells after induction of oxidative stress. The number shown in the panel indicates the number of cells quantified per genotype in the graph. (**C, D**) FACS analysis. (**C**) Representative flow cytometry dot plots show the intensity of TMRM signal in *DJ-1*−/− and +/+ MEFs following incubation with TMRM (50 nM) after incubation with or without H_2_O_2_ (500 µM, 3 hr) or pyocyanin (100 µM, 24 hr). (**D**) The bar graph shows quantification of TMRM signal in *DJ-1*−/− and +/+ MEFs. The TMRM fluorescence in *DJ-1*+/+ cells is decreased after incubation with oxidative stress inducers. The number shown in the panel indicates the number of embryos used to derive primary MEFs per genotype, and the data were obtained from three independent experiments. All data are expressed as mean ± SEM. *p<0.05, **p<0.01, ***p<0.001.

We then used similar approaches to determine the effects of oxidative stress inducers, such as H_2_O_2_ and pyocyanin, on mitochondrial transmembrane potential in *DJ-1*−/− and +/+ MEFs. We found that pretreatment of H_2_O_2_ (500 µM, 3 hr) or pyocyanin (100 µM, 24 hr) resulted in decreases in TMRM fluorescence in *DJ-1*+/+ MEFs (p<0.001, n = 15–28), compared to basal conditions (Fig. 10A and 10B). Quantitative analysis of TMRM fluorescence following FACS showed significant decreases of TMRM signals in *DJ-1*+/+ MEFs treated with H_2_O_2_ (p<0.05, n = 6) or pyocyanin (p<0.05, n = 6) (Fig. 10C and 10D). Treatment of H_2_O_2_ or pyocyanin eliminated the genotypic difference in mitochondrial membrane potential between *DJ-1*+/+ and *DJ-1*−/− MEFs (Fig. 10). These results indicate that increased oxidative stress results in marked reduction in mitochondrial membrane potential in *DJ-1*+/+ MEFs but has little effect on *DJ-1*−/− MEFs.

### Antioxidant Molecules Restore the mPTP Defect in *DJ-1−/−* MEFs and Oxidative Stress Inducers Increase mPTP Opening

**Figure 11 pone-0040501-g011:**
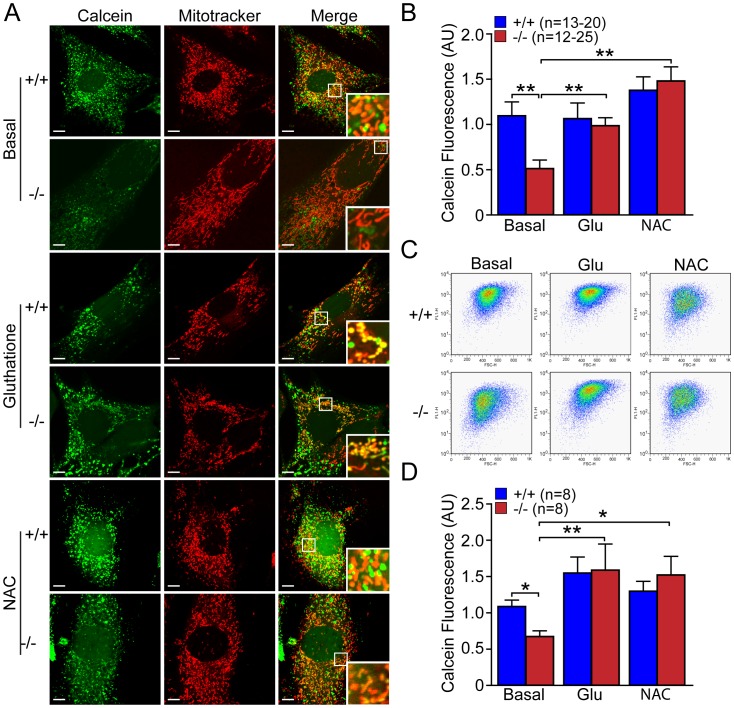
Antioxidants glutathione and NAC restore mPTP opening in *DJ-1*−/− MEFs. (**A, B**) Confocal microscopic analysis. (**A**) Representative confocal live cell images of *DJ-1*−/− and +/+ MEFs stained with calcein-AM (1 µM, green) and Mitotracker Red (150 nM) in the presence of Co^2+^ (1 mM) after incubation with or without glutathione (Glu, 10 mM, 24 hr) or NAC (20 mM, 24 hr). Insets show higher power views of the boxed area in the panel. Scale bar: 10 µm. (**B**) The bar graph shows quantification of calcein signal in *DJ-1*−/− and +/+ MEFs after incubation with or without glutathione (Glu, 10 mM, 24 hr) or NAC (20 mM, 24 hr). The calcein signal is reduced in *DJ-1*−/− cells under basal conditions, whereas this signal is increased in *DJ-1*−/− after incubation with antioxidants. The number shown in the panel indicates the number of cells quantified per genotype in the study. (**C, D**) FACS analysis. (**C**) Representative flow cytometric dot plots show the intensity of calcein signal in *DJ-1*−/− and +/+ MEFs following incubation with calcein-AM (1 µM) in the presence of Co^2+^ (1 mM) after incubation with or without glutathione (Glu, 10 mM, 24 hr) or NAC (20 mM, 24 hr). (**D**) The bar graph shows quantification of calcein signal in *DJ-1*−/− and +/+ MEFs and the reversal of the decrease of calcein fluorescence in *DJ-1*−/− cells after incubation with antioxidant molecules. The number shown in the panel indicates the number of embryos used to derive primary MEFs per genotype, and the data were obtained from three independent experiments. All data are expressed as mean ± SEM. *p<0.05, **p<0.01.

We then performed similar experiments to examine the effect of antioxidant molecules such as glutathione and NAC on mPTP opening. We treated *DJ-1*−/− and +/+ MEFs with glutathione (10 mM, 24 hr) or NAC (20 mM, 24 hr), and then measured calcein fluorescence using microscopic and flow cytometric analyses. Mitotracker Red (150 nM) was used as control for mitochondrial localization. Representative confocal live images (Fig. 11A) and quantification of calcein signal (Fig. 11B) showed that calcein fluorescence is increased in *DJ-1*−/− MEFs treated with glutathione (p<0.01, n = 12–25) or NAC (p<0.01, n = 12–25), relative to basal conditions. Quantitative analysis following FACS similarly showed increases of calcein fluorescence in *DJ-1*−/− cells after incubation with glutathione (p<0.01, n = 8) or NAC (p<0.05, n = 8), compared to basal conditions (Fig. 11C and 11D). Glutathione and NAC treatment did not have much effect on calcein fluorescence in *DJ-1*+/+ MEFs but eliminated the genotypic difference between *DJ-1*+/+ and *DJ-1*−/− MEFs (Fig. 11). These results showed that the increase in mPTP opening observed in *DJ-1*−/− cells is restored by antioxidant treatment.

**Figure 12:**
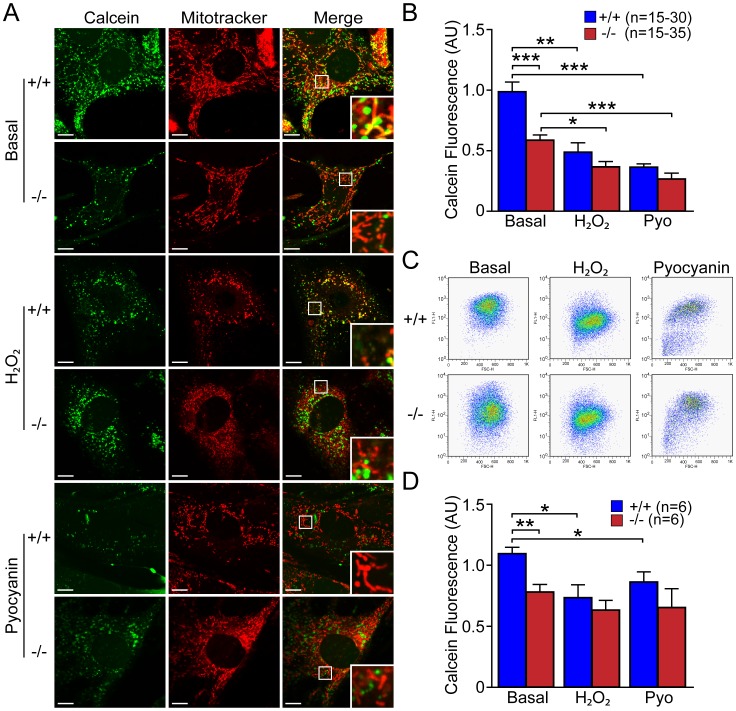
Increases of oxidative stress induce an increase in mPTP opening in ***DJ-1*** **+/+ MEFs.** (**A, B**) Confocal microscopic analysis. (**A**) Representative confocal live cell images of *DJ-1*−/− and +/+ MEFs stained with calcein-AM (1 µM, green) and Mitotracker Red (150 nM) in the presence of Co^2+^ (1 mM) after incubation with or without H_2_O_2_ (500 µM, 3 hr) or pyocyanin (100 µM, 24 hr). Insets show higher power views of the boxed area in the panel. Scale bar: 10 µm. (**B**) The bar graph shows quantification of calcein signal in *DJ-1*−/− and +/+ MEFs after incubation with or without H_2_O_2_ (500 µM, 3 hr) or pyocyanin (100 µM, 24 hr). Following treatment, the calcein signal is markedly reduced in *DJ-1*+/+ cells, and is also reduced in *DJ-1*−/− cells. The number shown in the panel indicates the number of cells quantified per genotype in the graph. (**C, D**) FACS analysis. (**C**) Representative flow cytometry dot plots show the intensity of calcein signal in *DJ-1*−/− and +/+ MEFs following incubation with calcein-AM (1 µM, green) in the presence of Co^2+^ (1 mM) after incubation with or without H_2_O_2_ (500 µM, 3 hr) or pyocyanin (100 µM, 24 hr). (**D**) The bar graph shows quantification of calcein signal in *DJ-1*−/− and +/+ MEFs. The calcein fluorescence in *DJ-1*+/+ cells is decreased after incubation with oxidative stress inducers. The number shown in the panel indicates the number of embryos used to derive primary MEFs per genotype, and the data were obtained from three independent experiments. All data are expressed as mean ± SEM. *p<0.05, **p<0.01, ***p<0.001.

We next evaluated the effect of ROS-inducing agents on mPTP opening in *DJ-1*−/− and +/+ MEFs using H_2_O_2_ (500 µM, 3 hr) or pyocyanin (100 µM, 24 hr). Representative confocal live images (Fig. 12A) and quantification of calcein signal (Fig. 12B) showed that calcein fluorescence is decreased in *DJ-1*+/+ MEFs in the presence of H_2_O_2_ (p<0.01, n = 15–35) or pyocyanin (p<0.001, n = 15–35). Quantitative FACS analysis of calcein fluorescence showed significant decreases of calcein signals in *DJ-1*+/+ MEFs treated with H_2_O_2_ (p<0.05, n = 6) or pyocyanin (p<0.05, n = 6), relative to basal conditions (Fig. 12C and 12D). *DJ-1*−/− MEFs treated with H_2_O_2_ (p<0.05, n = 15–35) or pyocyanin (p<0.001, n = 15–35) showed further decreases of calcein fluorescence in confocal analysis (Fig. 12A and 12B). These results further showed that increases of oxidative stress induce mPTP opening in primary MEFs.

## Discussion

Previously, we reported that loss of Parkin or PINK1 results in mitochondrial respiration impairment [27], [49], [50]. In the current study, we investigate whether inactivation of the third recessive PD gene, *DJ-1*, also affects mitochondrial respiration. Using primary MEFs and brains from *DJ-1*−/− mice, we found that endogenous respiratory activity as well as basal and maximal respiration are normal in intact *DJ-1*−/− MEFs, and substrate-specific state 3 and state 4 mitochondrial respiration are also unaffected in permeabilized *DJ-1*−/− MEFs and in isolated mitochondria from the cerebral cortex of *DJ-1*−/− mice (Fig. 1 and 2). Thus, in contrast to Parkin and PINK1, loss of DJ-1 does not affect mitochondrial respiration. However, mitochondrial transmembrane potential are reduced in the absence of DJ-1 (Fig.4), whereas mitochondrial permeability transition pore opening is increased (Fig. 5), though expression levels and activities of all individual complexes composing the electron transport system are unaffected (Fig. 3). Furthermore, ROS production is increased in *DJ-1*−/− MEFs (Fig. 7), and antioxidant treatment reverse the decreased mitochondrial transmembrane potential and the increased mitochondrial permeability transition pore opening in *DJ-1*−/− MEFs, whereas oxidative stress inducers have the opposite effects (Fig. 9–12). Together, these results suggest that DJ-1 regulates mitochondrial functions, such as mPTP opening and transmembrane potential, through its antioxidant role.

Earlier reports have demonstrated that DJ-1 functions as oxidative stress sensor and/or scavenger through oxidation of its conserved cysteine residues [11], [12], [14]. Mitochondria are the main site where ROS is produced in the cell, and excessive levels of ROS in mitochondria cause oxidization of all biomolecules, such as lipids, proteins and nucleic acids, leading to mitochondrial dysfunction. Consistent with these earlier reports, we confirmed that ROS production, measured by three different probes, is increased in the absence of DJ-1 (Fig. 7). In addition to being a ROS scavenger through its oxidation, other mechanisms of how DJ-1 may protect against oxidative stress have also been suggested. Superoxide dismutases (SOD1, SOD2), glutathione and catalase are essential defense mechanisms against oxidative stress in the cell, and DJ-1 has been reported to be involved in the glutathione metabolism and SOD1 expression [51]. Moreover it has been reported that DJ-1 is required for the transcription mediated by Nrf2 (nuclear factor erythroid 2-related factor), a master regulator of antioxidant transcriptional responses [52] and that DJ-1 influences the transcriptional activity of PGC-1α (peroxisome proliferator–activated receptor γ coactivator-1α), a transcriptional co-activator of a variety of genes including antioxidant genes and a master regulator of mitochondrial biogenesis [53]. However, we found that expression of antioxidative enzymes, such as catalase, G6PDH, SOD1 and SOD2 is normal in *DJ-1−/−* MEFs (Fig. 8).

Mitochondrial PTP opening is traditionally defined as a sudden increase of inner mitochondrial membrane (IMM) permeability due to the opening of a proteinaceous, voltage and Ca^2+^-dependent, and cyclosporin A (CsA)-sensitive permeability transition pore located in the IMM [54], [55]. The precise composition of the pore and regulatory mechanism of the pore opening are not fully understood, but evidence has indicted an involvement of mPTP in a number of pathological conditions including models of neurodegenerative diseases including PD [56], [57], [58], [59]. Under these pathological conditions, prolonged mPTP opening results in dissipation of Δ Ψ_m_, uncoupling of oxidative phosphorylation, failure to synthesize ATP and release of intramitochondrial Ca^2+^ and mitochondrial proteins such as cytochrome c or Apoptosis Inducing Factor (AIF), though whether these events trigger apoptotic or necrotic pathway remain controversial [54], [55]. Mitochondrial calcium and oxidative stress have been reported as major factors influencing mPTP opening [54], [55]. Our findings of unchanged mitochondrial calcium (Fig. 6) and increased ROS production (Fig. 7) in *DJ-1*−/− MEFs suggest that elevated ROS production likely underlies the increase in mPTP opening (Fig. 5), which in turn leads to decreased mitochondrial transmembrane potential (Fig. 4). The fact that the antioxidant treatment restores the defects in mPTP opening and mitochondrial transmembrane potential in *DJ-1*−/− MEFs (Fig. 9, 11) and that ROS-inducers have the opposite effects (Fig. 10, 12) provided additional support for this interpretation. Future studies will be needed to determine how elevated ROS production increases mPTP opening.

The most surprising result of the current study is the lack of mitochondrial respiration defects in the absence of DJ-1. Prior studies reported that mitochondrial respiration measured using the OROBOROS-oxygraph and Clark electrode system is reduced in immortalized *DJ-1*−/− MEFs [22] and in whole fruit flies lacking DJ-1 homologs but not in their heads [60]. However, using both primary MEFs and cerebral cortices from *DJ-1*−/− mice, we found that endogenous or substrate-induced respiratory activity is normal in *DJ-1*−/− MEFs, and that basal and maximal respiration measured using a more sensitive Seahorse Analyzer are also unaffected in the absence of DJ-1 (Fig. 1). Respiration in isolated mitochondria from the cerebral cortex of *DJ-1*−/− mice at 3 months or 2 years of age is also normal (Fig. 2), whereas loss of Parkin or PINK1 results in impaired mitochondrial respiration in similar experimental preparations [27], [49]. However, ATP production (Fig. 3) and mitochondrial transmembrane potential (Fig. 4) are reduced in all three experimental systems lacking DJ-1 [22], [60]. Prior reports have also shown that mitochondrial respiration defects and ATP production impairment are not always linked [49], [61], [62]. It will be interesting to understand the underlying mechanisms for this differential regulation.

These results suggest a divergence between DJ-1 and Parkin/PINK1 in the regulation of mitochondrial function. While inactivation of any of these recessive PD genes leads to mitochondrial function defects [22], [27], [49], [60], [63], [64], the specific defects differ. For example, mitochondrial calcium concentration is elevated in *PINK1*−/− neurons [50], [63], but levels of mitochondrial calcium in *DJ-1*−/− MEFs are unaffected (Fig. 6). Mitochondrial respiration is impaired in isolated mitochondria from *Parkin*−/− and *PINK1*−/− brains [27], [49], but it is normal in both intact and isolated mitochondria from *DJ-1*−/− mice (Fig. 1 and 2). Furthermore, ROS production is normal in *PINK1*−/− brains [49] but increased in *DJ-1*−/− cells (Fig. 7). However, mitochondrial permeability transition pore opening (Fig. 5) and mitochondrial transmembrane potential (Fig. 4) are similarly affected in *PINK1*−/− and *DJ-1*−/− mice [22], [50], [60], [63], [65], though the underlying mechanism appears to be different between *PINK1*−/− and *DJ-1*−/− mice with mitochondrial calcium dysregulation and overproduction of ROS being the initiating event, respectively. In summary, the current study on DJ-1 and our recent report on PINK1 [50] suggest that increased mPTP opening and the resulting change in mitochondrial transmembrane potential may be common mitochondrial defects shared by loss of these two PD gene products. Future studies will be needed to determine whether loss of Parkin causes increases in mPTP opening as well, and whether increases in mPTP opening render the cells lacking DJ-1, PINK1 and perhaps Parkin more vulnerable to induction of cell death.
